# Extrinsic factors can mediate resistance to BRAF inhibition in central nervous system melanoma metastases

**DOI:** 10.1111/pcmr.12424

**Published:** 2015-11-03

**Authors:** Heike Seifert, Eishu Hirata, Martin Gore, Komel Khabra, Christina Messiou, James Larkin, Erik Sahai

**Affiliations:** ^1^ Department of Medical OncologyRoyal Marsden NHS TrustLondonUK; ^2^ Tumour Cell Biology LaboratoryThe Francis Crick InstituteLondonUK; ^3^ Department of Oncologic PathologyKanazawa Medical UniversityKahoku‐gunIshikawaJapan

**Keywords:** metastatic melanoma, central nervous system metastases, brain metastases, vemurafenib, resistance, BRAF mutant

## Abstract

Here, we retrospectively review imaging of 68 consecutive unselected patients with BRAF V600‐mutant metastatic melanoma for organ‐specific response and progression on vemurafenib. Complete or partial responses were less often seen in the central nervous system (CNS) (36%) and bone (16%) compared to lung (89%), subcutaneous (83%), spleen (71%), liver (85%) and lymph nodes/soft tissue (83%), P < 0.001. CNS was also the most common site of progression.

Based on this, we tested in vitro the efficacy of the BRAF inhibitors PLX4720 and dabrafenib in the presence of cerebrospinal fluid (CSF). Exogenous CSF dramatically reduced cell death in response to both BRAF inhibitors. Effective cell killing was restored by co‐administration of a PI‐3 kinase inhibitor.

We conclude that the efficacy of vemurafenib is variable in different organs with CNS being particularly prone to resistance. Extrinsic factors, such as ERK‐ and PI3K‐activating factors in CSF, may mediate BRAF inhibitor resistance in the CNS.

**Significance**

Our clinical data show that the response to BRAF‐targeted therapy is highly variable dependent on the anatomical sites of metastases with CNS as a particular resistant site. We provide scientific evidence that BRAF inhibitor resistance in the CNS may be mediated by melanoma cell extrinsic factors in the cerebrospinal fluid. This requires further identification of those specific and potentially targetable factors in the future.

## Introduction

The selective BRAF inhibitors vemurafenib and dabrafenib are systemic treatments in patients with metastatic melanoma harbouring a V600 BRAF mutation, which accounts for roughly half cutaneous melanoma. BRAF V600 mutations activate the ERK/MAPK pathway, which plays an essential role in cell proliferation, differentiation and survival. Treatment with BRAF inhibitors results in high objective response rates, but progression occurs after an average of 6–7 months (McArthur et al., 2014).

Although extensively studied over the last few years, resistance mechanisms to BRAF‐targeted kinase inhibitors have not yet been fully understood (Bucheit and Davies, 2014). Multiple primary and acquired resistance mechanisms have been identified including those that lead to reactivation of the MAPK pathway and MAPK‐independent pathways, such as the PI3K/AKT/mTOR/cyclin D1/CDK4 retinoblastoma pathways (Bucheit and Davies, 2014). Melanoma cell intrinsic resistance to BRAF inhibitors seems to be diverse and independent resistance mechanisms may even develop in parallel in different tumour lesions (Chan et al., 2014; Menzies et al., 2014; Wilmott et al., 2012). Conversely, if progression occurs in one organ with ongoing response, in other organs, melanoma cell extrinsic factors may play a crucial role.

Patients with active metastatic central nervous system (CNS) disease were excluded from the initial registration trials of vemurafenib and dabrafenib and as a result, the efficacy of BRAF inhibitors in the CNS was uncertain and based on case reports. More recently, the results of phase II trials in metastatic melanoma patients with brain metastases demonstrated efficacy of both BRAF inhibitors vemurafenib and dabrafenib in the brain; however, progression‐free survival was short‐lived with approximately 4–6 months only (Azer et al., 2014; Dummer et al., 2014; Kefford et al., SMR 2013; Long et al., 2012). In patients with CNS metastases treated with dabrafenib, extra‐ and intracranial PFS was similar and there was little difference seen in efficacy between extra‐ and intracranial sites, but number of patients was limited (n = 23) and the analysis did not discriminate between sites of extracranial metastasis or the possibility that there was a larger initial number of extracranial metastases (Azer et al., 2014). Case reports have also reported solitary brain progression on vemurafenib with ongoing extracranial response, and different resistance mechanisms in the brain have therefore been suggested (Papadatos‐Pastos et al., 2013).

Here, we present organ‐specific efficacy and resistance data from a single‐institution retrospective analysis of BRAF V600‐mutant metastatic melanoma patients with progression on vemurafenib. We also provide experimental evidence that the poor responses of CNS metastases to vemurafenib and dabrafenib can be due to extrinsic factors present in cerebrospinal fluid (CSF).

## Results

### Clinical characteristics of the patient cohort

At time of evaluation, 68 patients treated with vemurafenib as a single agent for metastatic melanoma at our institution had stopped treatment due to radiologically confirmed progressive disease. Their pattern of progression is presented here. Baseline characteristics are summarized in Table 1. The majority of patients (69%) were treatment naïve before starting vemurafenib. The distribution of metastatic disease at baseline is presented in Table 2. The most common metastatic site was lymph nodes/soft tissue (81%). Metastatic CNS disease was present in 21% at baseline with a CT/MRI brain scan available in 81% of the patients. Thus, this group of patients reflects the normal metastatic pattern for melanoma.

**Table 1:** Baseline characteristics (n = 68)

Age, years (range)	53 (18–77)
Male, n (%)	39 (57)
Stage M1c, n (%)	55 (81)
Elevated LDH, n (%)	48 (71)
ECOG PS ≥ 2, n (%)	11 (16)
Systemic treatment prior to vemurafenib, n (%)	21 (31)
Number of metastatic organ sites at baseline	
1, n (%)	12 (18)
2, n (%)	11 (16)
≥3, n (%)	45 (66)

**Table 2:** Organ‐specific pattern of response and progression

	Baseline	Best response^a^		Progression			
		CR/PR	PD	Overall	Previous site	New site	Solitary site
	n (% total)	n (% BL)	n (% BL)	n (% total)	n (% BL (+))	n (% BL (−))	n (% total^b^)
Total, n (%)	68	47 (69)	9 (13)	68 (100)	65 (96)	20 (29)	20 (29)
LN/ST	55 (81)	45 (83)	5 (9)	35 (51)	35 (65)	–	9 (14)
Lung	35 (51)	31 (89)	–	16 (24)	14 (40)	2 (6)	2 (3)
Liver	33 (49)	28 (85)	3 (9)	19 (28)	18 (55)	1 (3)	2 (3)
Subcutaneous	29 (43)	24 (83)	–	14 (21)	13 (45)	1 (3)	–
Bone	19 (28)	3 (16)	3 (16)	13 (19)	8 (42)	4 (8)	2 (3)
CNS	14 (21)	5 (36)	2 (14)	19 (28)	11 (79)	6 (11)	4 (6)
Spleen	7 (10)	5 (71)	–	6 (9)	4 (57)	2 (3)	–
NOS	14 (21)	11 (79)	1 (7)	14 (21)	10 (71)	4 (7)	1 (2)

CR, complete remission; PR, partial response; PD, progressive disease; LN/ST, lymph nodes/soft tissue; CNS, central nervous system; NOS, not otherwise specified.

a 1 patient with CT head only to assess best response.

b 3 patients with CT head only at PD; BL (+) known metastatic disease in specific organ at baseline; BL (−) no metastatic disease in specific organ at baseline.

### Pattern of response and progression

**Figure 1:**
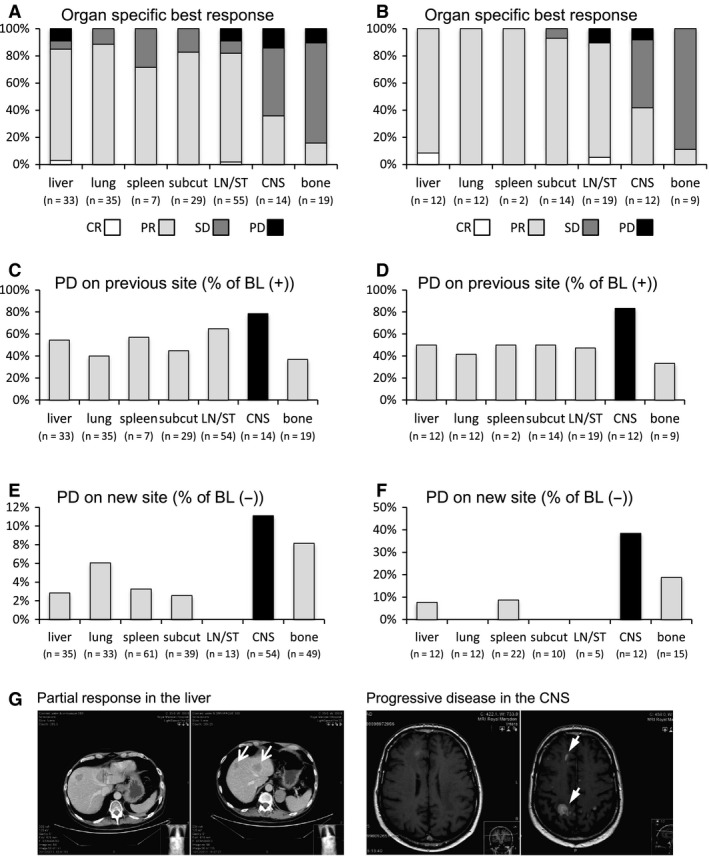
Organ‐specific response and progression pattern. (A) Organ‐specific best response in %. Fisher's exact test indicates that the disease response is significantly worse in the CNS and bones compared to lung, subcutaneous, spleen, liver and lymph nodes/soft tissue (P < 0.01). n, number of patients with organ‐specific metastatic disease at baseline and available CT scans for assessment; CR, complete remission; PR, partial remission; SD, stable disease; PD, progressive disease; subcut, subcutaneous; LN/ST, lymph nodes/soft tissue; CNS, central nervous system. (B) Similar to (A) except restricted to patients with CNS, abdomen and thorax imaging at both presentation and progression. (C) Organ‐specific progression (PD) at previous metastatic site: (n) number of patients with known metastatic site at baseline (BL (+)). (D) Similar to (C) except restricted to patients with CNS, abdomen and thorax imaging at both presentation and progression. (E) Organ‐specific progression (PD) on new sites: (n) number of patients with no previous metastatic site at baseline (BL (−)). (F) Similar to (E) except restricted to patients with CNS, abdomen and thorax imaging at both presentation and progression. (G) Imaging of a patient with multiple intraparenchymal brain metastases at baseline (left side of right panel) with evidence of several liver metastases (left side of left panel). After 2 months of treatment with vemurafenib, the patient showed clinical symptomatic progression in the CNS with several new brain metastases (right side of right panel), but response in the liver (right side of left panel).

Median time on vemurafenib until progression was 4.1 months (95% CI: 0.9–17.1). Overall, one patient (1%) on vemurafenib showed complete remission (CR), 68% partial response (PR), 18% SD and 13% progressive disease (PD) as best response. We assessed whether the responses were equivalent at different metastatic locations. As shown in detail in Figure 1(A) and Table 2, CR or PR was more common in metastatic disease in the lung (89%), liver (85%), lymph nodes/soft tissue (83%), subcutaneous (83%) and spleen (71%), whilst they were less common in the CNS (36%) and bones (16%), P < 0.01 by Fisher's exact test. Primary refractory disease was most common in bone and CNS disease (16 and 14%, respectively). Complete imaging (CNS/thorax/abdomen/pelvis) at baseline and progression was available in 24 patients and confirms poorer responses in CNS and bone (Figure 1B).

At progression, in 65 of 68 patients, a CT thorax/abdomen/pelvis was available for review, whilst in three patients, only CT head was performed. This enabled us to evaluate whether progression was equally common in different organs. Progression in 1, 2 or ≥3 organ sites was seen in 29, 43 and 24% of the 68 patients, respectively. The most common site of progression on vemurafenib was the CNS; 79% of the patients with previously known CNS metastases progressed in the CNS. This contrasts with 40, 45, 55, 57 and 65% progression of lung, subcutaneous, liver, spleen and lymph node/soft tissue metastases, respectively (Figure 1C). The same pattern was even more evident when analysis is restricted to the patients with complete imaging (CNS/thorax/abdomen/pelvis) at baseline and progression (Figure 1D). Further, the CNS was the most common site for new metastases to arise (Figure 1E,F). Patients with progression within the CNS showed a trend to poorer OS compared to those without (median OS 6.0 months (95% CI: 2.1–9.9) versus 9.0 (95% CI: 7.5–10.5), P = 0.862).

### Progression pattern in central nervous system disease

With CNS being the most common site of progression, we further assessed the pattern of response and progression in the CNS in detail. Of 14 patients with CNS disease at baseline, 11 patients had parenchymal brain lesions, two patients had meningeal disease, and one patient had both parenchymal and meningeal disease. One patient had received prior CyberKnife treatment on two parenchymal lesions, one patient had received whole‐brain radiotherapy (WBRT) for one lesion, and another patient had WBRT for multiple lesions. In these 14 patients, PR, SD and PD were seen in 5 (36%), 7 (50%) and 2 (14%) patients, respectively (Table S1). In four of the five patients with response to vemurafenib, only a solitary brain metastasis was present at baseline, while one patient presented with meningeal disease. None of these patients had received brain radiotherapy prior to vemurafenib. Amongst the patients with stable CNS disease (parenchymal n = 5, meningeal n = 1, both n = 1), only two patients showed more than one parenchymal metastases and they received CyberKnife or WBRT prior to vemurafenib. Ultimately, both of these patients showed progression on the irradiated sites. Both patients with primary refractory CNS disease presented with multiple brain metastases at baseline, but had not received WBRT before starting vemurafenib.

At cessation of vemurafenib, 19 of 68 patients (28%) showed progression within the CNS with a CT thorax/abdomen/pelvis available in 18 patients. Twelve of these patients (66%) showed synchronous progression within and outside the CNS. CNS progression only was seen in four of the 18 patients (22%) including one patient with meningeal disease. Images of a patient with intracranial disease progression, but ongoing extracranial response, are shown in Figure 1G. Progression outside the CNS with ongoing response within the CNS was seen in two patients with an asymptomatic solitary small volume brain metastasis at baseline.

### In vitro analysis of a potential extrinsic resistance mechanism of BRAF inhibitors in the central nervous system

Based on the analysis above, we hypothesized that there may be some distinctive feature of the CNS environment responsible for the poor responses to vemurafenib. Cerebrospinal fluid contains various growth factors and cytokines, some of which are reported to activate ERK/MAP kinase signalling in a Ras‐dependent manner (Bilic et al., 2006; Lok et al., 2014). Thus, we decided to explore the direct effect of CSF on the response to BRAF inhibition. We established a BRAF‐mutant melanoma cell line expressing an ERK/MAP kinase ‘biosensor’ (Hirata et al., 2015; Komatsu et al., 2011). The biosensor is an engineered polypeptide that contains two fluorophores and can be phosphorylated by ERK/MAP kinase. This phosphorylation event leads to a conformational change in the polypeptide that repositions the fluorophores and alters the fluorescent properties of the molecule. The change in fluorescence can be monitored by live cell microscopy, thereby providing a dynamic read‐out of ERK/MAP kinase activity in BRAF‐mutant melanoma cells.

**Figure 2:**
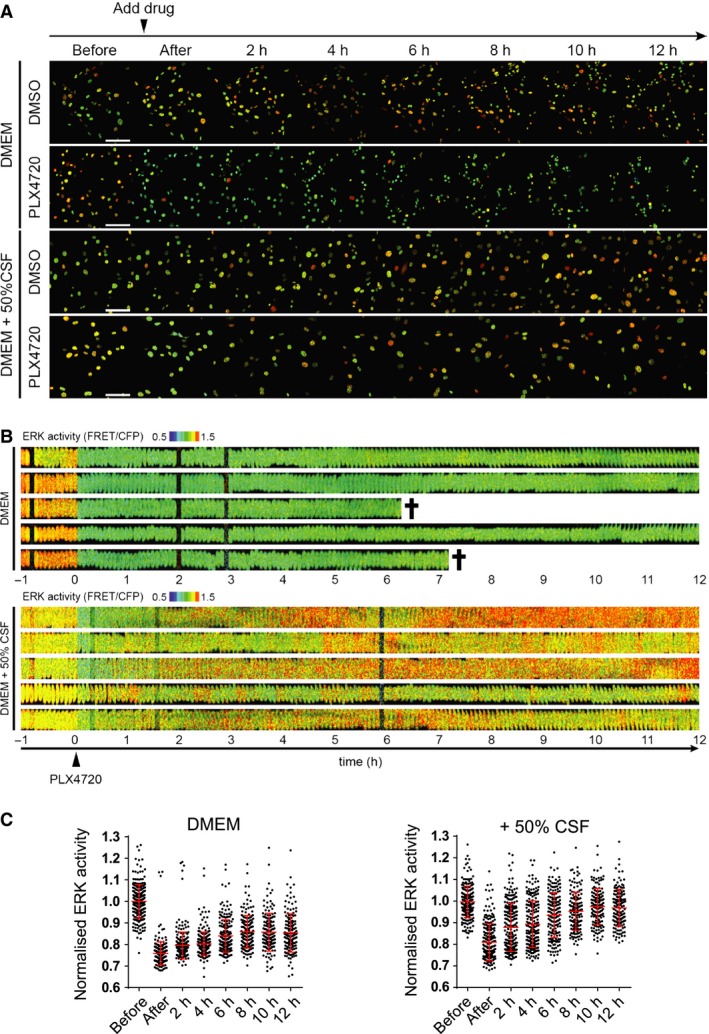
Cerebrospinal fluid (CSF) counteracts the ability of PLX4720 to reduce ERK/MAP kinase activity. (A) Time‐lapse images of 5555 mouse melanoma cells stably expressing EKAREV‐NLS biosensor cultured in DMEM or DMEM containing 50% rat CSF, treated with 0.1% DMSO or 1 μM PLX4720 (at time 0, indicated by an arrowhead). Images were acquired every 5 min for 13 h. See also Movie S1. Scale bar = 100 μm. (B) Kymographs showing ERK/MAPK biosensor FRET activity of five representative cells either treated with 1 μM PLX4720 in DMEM (upper set) or 1 μM PLX4720 in 50% CSF (lower set). Black crosses indicate cell death. (C) Quantification of ERK/MAPK biosensor activity of 5555 cells treated with PLX4720 in DMEM (top) or DMEM + 50% CSF (bottom). Each dot in the graphs represents ERK activity in a single cell, normalized to before treatment.

The upper panel in Figure 2(A) shows the baseline activity of ERK/MAP kinase in murine BRAF‐mutant melanoma cells (Dhomen et al., 2009). This is somewhat heterogeneous but does not change during 12 h of live cell imaging. In contrast, if the BRAF inhibitor PLX4720 is added after 5 min of imaging, then there is a sharp reduction in ERK/MAP kinase activity (this is visible as a ‘blue shift’ in the false colour scale in Figure 2A–C). The reduction in ERK/MAP kinase activity persists for several hours until the melanoma cells begin to die (movie 1 and quantified in Figure 2).

**Figure 3:**
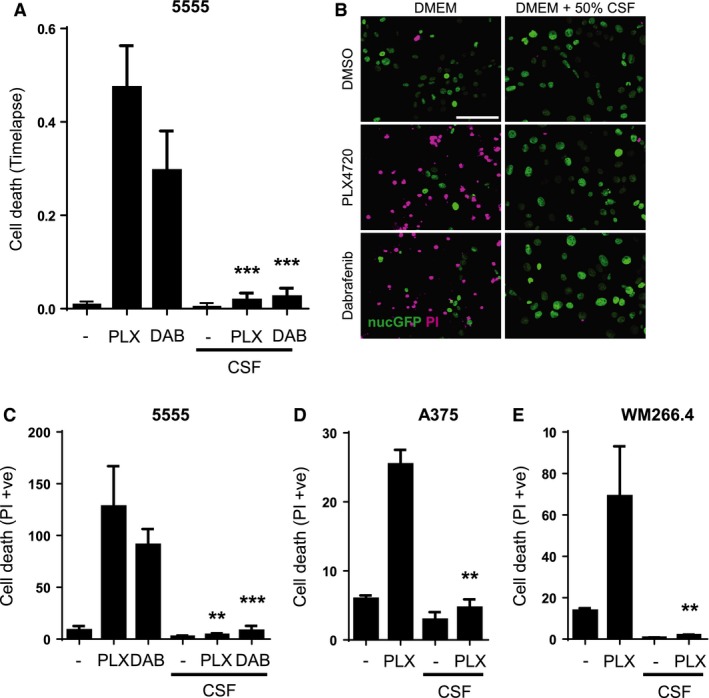
Cerebrospinal fluid (CSF) counteracts the ability of BRAF inhibitors to induce cell death. (A) Histogram shows the cell death of 5555 cells cultured in DMEM or 50% CSF treated with DMSO, 1 μM PLX4720 or 1 μM dabrafenib. ***P < 0.001. The percentage of cell death observed in each treatment group during a 12‐h movie was quantified (unpaired *t*‐test, n = 4). (B) Images show cell death of 5555 cells cultured in DMEM or 50% CSF treated with DMSO or 1 μM PLX4720 or 1 μM dabrafenib. Propidium iodide staining is shown in magenta, and nuclear GFP staining is shown in green. Scale bar is 100 μm. (C) Quantification of cell death induced by 1 μM PLX4720 or 1 μM dabrafenib in 5555 cells cultured in DMEM or 50% CSF (n = 3). **P < 0.01, ***P < 0.001. (D) Quantification of cell death induced by 1 μM PLX4720 in A375 cells cultured in DMEM or 50% CSF (n = 3). **P < 0.01. (E) Quantification of cell death induced by 1 μM PLX4720 in WM266.4 cells cultured in DMEM or 50% CSF (n = 3). **P < 0.01.

We next investigated what happens if melanoma cells are cultured in the presence of CSF isolated from rats. In the presence of CSF but absence of PLX4720, the activity of ERK/MAP kinase is similar to controls but marginally increases during the 12‐h imaging session. The addition of PLX4720 causes a reduction in ERK activity after 5 min in both control and CSF cultures. However, in the presence of CSF, the reduction in ERK/MAP kinase activity is short‐lived, and after 12 h, is undistinguishable from that prior to PLX4720 addition. This demonstrates that exogenous CSF dramatically shortens the kinetics of BRAF‐mutant melanoma cell response to PLX4720. This is correlated with a failure of the melanoma cells to die (movie 1 and quantified in Figure 3A). We confirmed that CSF provides protection against the effect of BRAF inhibition using a second drug, dabrafenib (Figure 3A). The apoptotic nature of PLX4720‐induced cell death and its reduction by exogenous CSF were confirmed by live staining using cell viability markers. Both propidium iodide and annexin V staining confirmed the ability of CSF to counteract the effects of BRAF inhibition (Figure 3B,C and Figure S1A). Further, the detection of annexin V‐positive cells that retained nuclear integrity confirmed the early externalization of phosphatidylserine, which is a hallmark of apoptotic cell death (yellow arrow in Figure S1A). We sought to validate our observations about the protective effect of CSF in models of human V600‐mutant melanoma. Both A375 and WM266.4 human melanoma cell lines harbour BRAF mutations and are sensitive to PLX4720 (Figure 3D,E). The effect of PLX4720 is reduced in both cell lines by adding exogenous CSF. We additionally confirmed that CSF prevents PLX4720 from reducing overall cell numbers (Figure S1A,B). These data demonstrate that CSF reduces the efficacy of BRAF inhibition in multiple models, including human melanoma cells.

**Figure 4:**
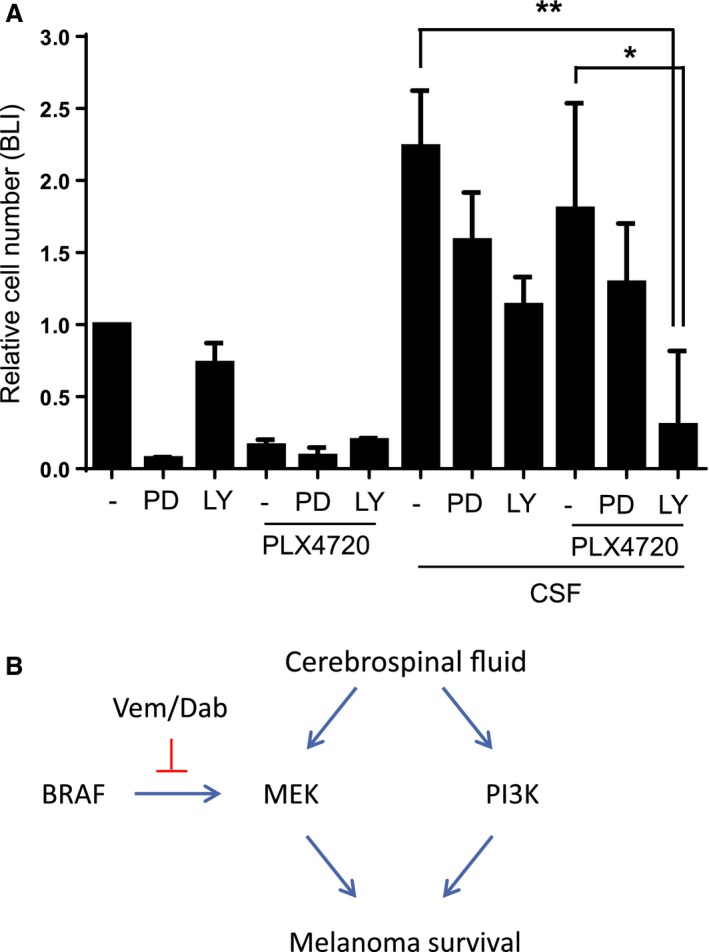
BRAF and PI‐3 kinase inhibitions synergize to reduce cell viability in the presence of cerebrospinal fluid (CSF). (A) Histogram shows the cell viability of 5555 luciferase cells cultured in DMEM or 50% CSF treated with DMSO, 1 μM PLX4720, 1 μM PD184352 or 10 μM LY294002. Cell viability was measured by assessing luciferase activity (n = 4). *P < 0.05, **P < 0.01. (B) Model of BRAF inhibition in the presence of CSF: scheme shows how CSF can activate ERK/MAPK signalling in a BRAF‐independent manner and PI‐3 kinase signalling. These events combine to reduce the efficacy of BRAF inhibitors in the presence of cerebrospinal fluid.

We next sought to understand which signalling pathways might be mediating the protective effect of CSF. We hypothesized that either the re‐activation of ERK/MAP kinase signalling or PI‐3 kinase signalling may be important; the latter can be activated by factors in CSF such as IGF‐1 and IL‐6 (Bilic et al., 2006; Protas et al., 2011). To test the functional significance of these pathways, we combined PLX4720 treatment with either a MEK inhibitor, PD184352 or a pan PI‐3 kinase inhibitor, LY294002. Figure 4A shows that combined blockade of BRAF and PI3K isoforms synergizes to reduce melanoma cell number in the presence of CSF. Interestingly, CSF provided significant protection against the effect of either MEK inhibition alone or combined BRAF and MEK inhibition. These data indicate that the protective effects of CSF are largely mediated via PI‐3 kinase signalling.

## Discussion

Our clinical data demonstrate that vemurafenib shows efficacy in all organs and this concurs with the observation of others (Dummer et al., 2014; Kefford et al., SMR, 2013), but the efficacy appears highly variable depending on anatomical sites. We found that lymph node/soft tissue, lung, liver, spleen or subcutaneous metastases respond better than either CNS or bone metastases. Moreover, the CNS was also the most common site where resistance emerged during treatment with vemurafenib. The different responses seen in the bone and CNS were highly significant. These patterns of response hold true for both the analysis of all 68 patients and the 24 patients for whom complete imaging at both presentation and progression was available. Although difficulties can arise in the unambiguous assessment of disease in the bone and CNS and only contrast CT scan without MRI was available at baseline, these are highly unlikely to account for the pattern of response that we observe. We suggest, therefore, that the CNS and bones represent an environment, where organ‐specific extrinsic factors may play an important role in BRAF inhibitor resistance. The poor responses in the bone are consistent with our recent findings that stiff microenvironments rich in matricellular molecules enable BRAF‐mutant melanoma to tolerate BRAF inhibition (Hirata et al., 2015). Our findings are consistent with a previous report that the outcome of CNS disease is poor (Meier et al., 2004).

As demonstrated in the published results of the phase II trials of single‐agent vemurafenib or dabrafenib in patients with untreated and treated brain metastases (Dummer et al., 2014; Kefford et al., SMR, 2013; Long et al., 2012), our data confirm efficacy of vemurafenib in the CNS, but secondary resistance to vemurafenib was almost universal. Only patients with low volume CNS disease showed response in our study; all patients with multiple brain metastases showed primary refractory disease. Interestingly, 4 patients showed CNS progression only with ongoing response outside the CNS. Without detailed analyses of biopsies from both responding and non‐responding metastases in the same patient, it remains unclear whether primary resistant CNS disease or solitary CNS progression is due to melanoma cell intrinsic mechanisms or extrinsic factors. Nonetheless, the pattern of poor responses in the CNS across the whole group of patients suggests that extrinsic factors may play a role.

Preclinical data suggest limited efficacy of BRAF inhibitors within the CNS due to active drug efflux transporters such as P‐glycoprotein (ABCB1) and breast cancer resistance protein (BRCP or ABCG2) (Mittapalli et al., 2013). Other explanations so far are the limited penetration of the blood–brain barrier due to the chemical characteristics of the BRAF inhibitor vemurafenib with relatively large molecular weight and poor lipid solubility (Wager et al., 2011). Inflammatory changes of the blood–brain barrier, such as caused by WBRT, are known to allow greater drug penetration. However, in the phase II trial with the BRAF inhibitor dabrafenib and vemurafenib, no difference between patients with metastatic brain disease with or without prior local treatment (surgery or radiotherapy) was seen in the outcome (Kefford et al., SMR, 2013; Long et al., 2012). Therefore, restricted permeation of BRAF inhibitors into the CNS may not be the most important limitation in efficacy.

In our in vitro tests, exposure of PLX4720‐sensitive melanoma cells to cerebrospinal fluid resulted in an upregulation of ERK signalling. Thus, the presence of high levels of ERK‐activating factors in the CSF/CNS may explain one potential extrinsic source for BRAF inhibitor resistance in the CNS. However, combined blockade of BRAF and MEK was relatively ineffective in the presence of CSF and cotargeting of BRAF PI‐3 kinase was much more effective. This implies a significant role for PI‐3 kinase signalling triggered by CSF, which is consistent with the work of Chen and colleagues (Chen et al., 2014). Further, higher activation of the AKT survival pathway has been demonstrated in astrocyte‐conditioned medium compared to fibroblast‐conditioned medium and inhibition of PI3K/AKT signalling resensitized melanoma cells isolated from a vemurafenib‐resistant brain metastasis (Niessner et al., 2013). In the future, it will be interesting to identify the specific factors within CSF that promote ERK activity and determine whether they might be therapeutically targeted. Indeed, high levels of IGF‐1 have been reported in CSF and its receptor would represent a ‘druggable’ target (Pyonteck et al., 2013). Recent preclinical data showed potent activity of MEK/ERK inhibitors (Carlino et al., 2014;). But limited brain distribution was demonstrated in MEK inhibitors (Vaidhyanathan et al., 2014) and the clinical benefit of MEK inhibitors in patients with CNS metastases is currently investigated in an ongoing trial (COMBI‐MB – NCT02039947 (2015)). Our data argue that even combined inhibition of BRAF and MEK may have limited efficacy against brain metastases.

To conclude, our analysis of melanoma response to vemurafenib depending on anatomical location reveals that CNS disease is particularly problematic. We propose that cell extrinsic factors in the CNS environment provide a BRAF‐independent mechanism for ERK/MAP kinase activation. We demonstrate experimentally that CSF greatly diminishes the responsiveness of BRAF‐mutant melanoma cells to PLX4720. In the future, it will be interesting to perform more detailed analyses of biopsies from different locations in the same patient to determine the role of cell intrinsic versus extrinsic factors in the varying response of disease at different sites. Greater understanding of the mechanisms of organ‐specific influences on metastases, and how to counteract them, should lead to improvements in the utility of available targeted therapies in metastatic melanoma.

## Methods

### Clinical and imaging review

Retrospectively, we analysed all patients that had been treated with and progressed on vemurafenib as a single agent for metastatic melanoma harbouring a BRAF V600 mutation at a single institution (Royal Marsden Hospital, London, UK) from March 2010 to May 2013. Patients were treated within the BRIM‐3 study (NCT01006980), the vemurafenib safety study (NCT01307397) as well as off trial after the European Medicines Agency (EMA) approval of vemurafenib in December 2011. Exclusion criteria were as follows: cessation of vemurafenib for reasons other than progressive disease, for example toxicity, and lack of radiological confirmation of progressive disease. Contrast CT/MRI scans at baseline, during treatment and at progression were assessed for overall best response per Response Evaluation Criteria in Solid Tumours (RECIST) version 1.1. Organ‐specific response was assessed using a modified form of RECIST 1.1 criteria: any reduction in target lesions was assessed as PR, stable appearance was defined as stable disease (SD), and growth of ≥20% in one lesion or the sum of the organ lesions or any new lesion was defined as primary refractory disease if present on first evaluation scan or as PD on subsequent scans. The regulatory oversight of this study was by the Royal Marsden Hospital/Institute of Cancer Research Committee for Clinical Research.

### In vitro analyses

#### Cells, probes and reagents

BRAF‐mutant 5555 mouse melanoma cells are described in Manning et al. (2013, 2015) and were a kind gift from Professor Richard Marais (The CRUK Manchester Institute, UK). A375 and WM266.4 human melanoma cell lines were a gift from Professor Chris Marshall (Institute of Cancer Research, London, UK). Melanoma cells were maintained in DMEM supplemented with 10% FBS (Dhomen et al., 2009). The prototype FRET biosensors for ERK/MAPK (EKAREV‐NLS) were described previously and are a kind gift from Professor Michiyuki Matsuda (Kyoto University, Japan) (Komatsu et al., 2011). The probe was introduced into the cells with PiggyBac transposon system (Wellcome Trust Sanger Institute, UK). PLX4720 was obtained from Stratech (#S1152) and used at the final concentration of 1 μM. The COSHH and genetic modification regulatory oversight of this work was performed by the London Research Institute Health and Safety Committee.

#### Collection of rat cerebrospinal fluid

Rat CSF was collected from male Wistar rats as described elsewhere (Liu and Duff, 2008). Briefly, animals were euthanized by overdose carbon dioxide and fixed on a surgical stage. After dissecting occipital muscle layers, CSF (~150 μl/rat) was directly collected with a microsyringe by punctuating Cisterna Magna. Collected CSF was aliquoted and kept at ‐80⁰C until experimental use.

#### Time‐lapse FRET imaging and image processing

5555‐EKAREV‐NLS were seeded onto a glass bottom 96‐well plate and cultured in 100 μl phenol red‐free DMEM (GIBCO/Thermo Fisher Scientific, Waltham, MA, USA) or mixture of 50 μl phenol red‐free DMEM and 50 μl rat CSF. For the dual‐emission ratio imaging, we used Zeiss 780 inverted microscope with 20× dry objective (Nikon Instruments Europe B.V., Amsterdam, Netherlands). The FRET biosensor was excited by a Chameleon Ti: Sapphire Laser (Coherent Inc., Cambridgeshire, UK) at 820 nm excitation wavelength through IR cut filter (MBC 760). The emission light was separated by beam splitters into 463–506 nm for CFP and 515–559 nm for YFP/FRET. Images were acquired as 12‐bit images and analysed with metamorph software (Universal Imaging), as described previously (Hirata et al., 2012).

#### Cell viability assay

A total of 25 000 melanoma cells were seeded on 96‐well glass bottom plates, cultured overnight and then treated with DMSO (0.4%), PLX4720 (1 μM), dabrafenib (1 μM), PD184352 (1 μM), LY294002 (1 μM) or the combinations for 24 h. For cell death analysis, melanoma cells stably expressing YFP in the nuclei were stained with propidium iodide (PI) at the final concentration of 1 mg/ml and imaged with Zeiss 780 inverted microscope. Then, PI‐positive areas were calculated per the number of surviving cells. For cell proliferation assay, melanoma cells stably expressing firefly luciferase were seeded and treated in the same manner, and after adding D‐Luciferin (150 μg/ml), the plates were imaged under IVIS Spectrum (Parkin Elmer).

## Conflict of interest

James Larkin has consulting and advisory role for BMS, MSD, GSK, Pfizer, Novartis, Roche/Genentech and receives research funding from Novartis and Pfizer. Martin Gore participated in speakers’ bureaux for Roche. All remaining authors have declared no conflict of interests.

## Supporting information


**Figure S1.** (A) Images show cell death of 5555 cells cultured in DMEM or 50% CSF treated with DMSO or 1 *μ*M PLX4720 or 1 μM dabrafenib. Annexin V staining is shown in magenta and nuclear GFP staining is shown in green. White arrow highlights cell in the early stages of apoptosis – nuclear morphology normal but annexin V +ve, yellow arrow highlights cell in later stages of apoptosis – abnormal nuclear morphology and annexin V +ve, orange arrow highlights apoptotic cell debris – histone H1 is no longer visible but annexin V staining can still be seen associated with the cell membrane. Scale bar is 100 μm. Histogram shows Quantification of cell death induced by 1 μM PLX4720 or 1 μM dabrafenib in 5555 cells cultured in DMEM or 50% CSF (n = 6 fields of view from two experiments). ***P < 0.001. (B) Histogram shows the cell viability of A375‐luciferase cells cultured in DMEM or 50% CSF treated with DMSO or 1 μM PLX4720. Cell viability was measured by assessing luciferase activity (n = 3). **P < 0.01. (C) Histogram shows the cell viability of WM266.4‐luciferase cells cultured in DMEM or 50% CSF treated with DMSO or 1 μM PLX4720. Cell viability was measured by assessing luciferase activity (n = 3). **P < 0.01.

**Movie S1.** CSF provides an extrinsic factor that reactivates ERK in PLX4720 treated melanoma cells.
